# Resistin mediates tomato and broccoli extract effects on glucose homeostasis in high fat diet-induced obesity in rats

**DOI:** 10.1186/s12906-016-1203-0

**Published:** 2016-07-18

**Authors:** Nora M. Aborehab, Mahitab H. El Bishbishy, Nermien E. Waly

**Affiliations:** Department of Biochemistry, Faculty of Pharmacy, MSA University, Giza, 11787 Egypt; Department of Pharmacognosy, Faculty of Pharmacy, MSA University, Giza, 11787 Egypt; Department of Physiology, Faculty of Medicine, Helwan University, Helwan, 11787 Egypt

**Keywords:** Tomato, Broccoli, Glucose homeostasis, Resistin, Obesity

## Abstract

**Background:**

Resistin is an adipocyte hormone that regulates glucose metabolism. Elevated levels of resistin may cause insulin resistance. This may link obesity, and increased fat mass to type II diabetes and insulin resistance. We hypothesized that treatment with tomato and broccoli extracts regulates glucose homeostasis via modulation of resistin levels in high fat diet-induced obesity rats (HFD).

**Methods:**

Forty-eight male albino rats were divided into 8 groups as follows: control, HFD, stop fat diet (SD), Tomato 200 mg/kg (T200), Tomato 400 mg/kg (T400), Broccoli 200 mg/kg (B200), Broccoli 400 mg/kg (B400), and Chromax (CX). Treatment continued for 1 month. Serum levels of resistin, leptin, adiponectin, glucose and insulin were measured using ELISA and spectrophotometry.

**Results:**

Serum levels of resistin were significantly reduced in the T 200, T 400, B 200, B 400 and CX groups to: 4.13 ± 0.22 ng/ml, 1.51 ± 0.04 ng/ml, 4.13 ± 0.22 ng/ml, 2.32 ± 0.15 ng/ml and 1.37 ± 0.03 ng/ml, respectively, compared to HFD group and SD group (*P* value < 0.0001). Non-significant differences were found between T 400, B 400 and CX groups. Serum levels of leptin were significantly reduced in the T 400 (22.7 ± 0.84 pg/ml) group compared to the B 400 (41 ± 2.45 Pg/ml) and CX groups (45.7 ± 2.91 Pg/ml), *P* value < 0.001. Serum levels of adiponectin were significantly increased in the T 400 group (131 ± 3.84 pg/ml) compared to the CX group (112 ± 4.77 pg/ml), *P* value < 0.01.

**Conclusions:**

Our results demonstrate that tomato and broccoli extract treatment regulates glucose homeostasis via reduction of serum resistin and may be a useful non-pharmacological therapy for obesity.

**Electronic supplementary material:**

The online version of this article (doi:10.1186/s12906-016-1203-0) contains supplementary material, which is available to authorized users.

## Background

Obesity is an escalating global and societal problem. Associated environmental factors such as poor dietary habits, sedentary lifestyle, socioeconomic influences; and genetic disorders that affect hormone secretion and metabolism result in weight gain [1]. Western-style diets, low in dietary fiber and high in saturated fatty acids, are implicated in increased risk of diabetes and obesity [2]

Adipose tissues are now considered to actively regulate the pathways responsible for energy balance. A complex network of hormonal and neuronal signals controls the activity of these pathways. Adipocytes secrete chemical substances called adipocytokines like leptin, adiponectin, resistin, tumor necrosis factor (TNF)-α, interleukin-6, and angiotensinogen [3].

Resistin was identified as an adipocyte hormone that participates in the regulation of glucose metabolism, increasing blood glucose levels and hepatic glucose production. Elevated levels of resistin are postulated to cause insulin resistance. This may link obesity, and increased fat mass to type 2 diabetes and insulin resistance [4].

There is a continuous effort among researchers and dieticians to identify food items or dietary components that may prevent or treat obesity and its complications. It was reported that tomato (*Solanum lycopersicum* L., Family Solanaceae), might serve as a functional food that has beneficial effects on health other than nutrition [5]. These effects include but are not limited to anti-platelet, anti-oxidant and anti-cancer effects [6, 7]. Tomato was also found to inhibit the cancer cell proliferation induced by some estrogens [8, 9]. Tomato extract was effective in protecting against liver inflammation induced by a high fat diet (HFD)-by decreasing both the number of inflammatory foci and the expression of multiple pro-inflammatory cytokines [10].

Broccoli (*Brassica oleracea L. Italica*, Family Brassicaceae) is another candidate of functional foods. Broccoli might be beneficial in reducing the risk for the development of certain forms of cancer as well as its antioxidant effect [11–13]. It was also recently found that broccoli extract was found to increase glucose uptake and induce lipogenesis in cell culture [14]. Also, broccoli fiber and high dietary corn oil resulted in lower serum cholesterol and triglycerides, which may have beneficial effects on lipid metabolism [15].

In this study we investigated the potential role of aqueous extracts of tomato and broccoli grown in Egypt in regulating blood glucose level in HFD-induced obesity in rats, as well as the possible role of resistin in maintenance of glucose homeostasis. We also studied the nutritional values of these foods to assess their potential use as functional foods.

## Methods

### Plant collection and preparation of extracts

#### Plant material

Ten kilograms of the fresh stalks and flower heads of broccoli (cultivar Snowball) were collected from the El Orman botanical garden, Giza, Egypt. Nine kilograms of the fresh fruits of Tomato (cultivar Peto 86) were collected from the National Research Center Agricultural Station, Nubaria, Behera Governorate, Egypt. The taxonomical identity was kindly verified by Dr. Mohamed El Gebaly, National Research Center, Giza, Egypt. The material was collected during 2015. The fresh plant material was used for the phytochemical investigations. A voucher specimen was deposited in the herbarium of The Faculty of Pharmacy, Modern Science and Arts University (MSA).

#### Extract preparations

The plant materials were separately grinded then macerated in distilled water for 24 h. The aqueous extracts were concentrated under reduced pressure and kept in tightly closed amber glass containers for biochemical analysis.

Two doses of 200 mg/kg and 400 mg/kg were selected and used in this study for both tomato and broccoli extracts [9]. The two doses were prepared by dissolving 0.5 or 1 mg of this extract into 10 ml distilled water [16].

#### Reference material and solvents

Authentic vitamins (A, B1, B2, B3, B6, B12, C and E) were kindly supplied by the Agricultural Research Center, Food Technology Research Institute, Giza, Egypt. All chemical reagents and solvents were analytical grade.

### Phytochemical Investigations

#### Proximate analysis

Proximate analysis of tomato and broccoli fresh samples included the determination of their nutritive value, moisture content, total ash, total carbohydrates, total lipids and total proteins. The nutritive values were determined by multiplying the values obtained for protein, fat and carbohydrate by 4, 9 and 4, respectively and adding up the values [17]. The moisture contents, total ash content, total protein and lipid extraction were determined according to A.O.A.C guidelines [18–20]. Total carbohydrates were determined according to (Dubois et al. 1956 [21].

#### Analysis of the mineral content

The mineral content was determined according to A.O.A.C guidelines [19]. Samples were separately digested in an Advanced Microwave Digestion System ETHOS 1 by wet digestion with concentrated sulfuric acid in the presence of digestion catalysts (a mixture of copper sulfate and anhydrous sodium sulfate, 1:10), then the resulted solutions were measured using an Atomic Absorption Spectrometer (Inductively Coupled Plasma ICP-AES Spectrometer, iCAP 6000 series, Thermo Scientific).

#### Determination of vitamins by HPLC

A Hewlett-Packard Series 1,100 liquid chromatography system (Waldbronn, Germany) equipped with a loop (20 μl) diode array detector and a lichrosorb RP 15 column (4.0 mm i.d.x 250 mm; particle size 5 mm) (Merck, Darmstadt) was used for vitamin content determination. Elution was performed at a flow rate of 1.0 ml/min with a mobile phase of water/acetic acid (98: 2 v/v, solvent A) and methanol/acetonitrile (50: 50, v/v, solvent B), starting with 5 % B and increasing B to levels of 30 % at 25 min, 40 % at 35 min, 52 % at 40 min, 70 % at 50 min, 100 % at 55 min, and kept at this stage for 5 min. A re-equilibration time of 15 min was then required. Quantitation was achieved at 280 nm by an internal standard method [22]. Vitamins A and E contents were determined according to the method described by Beyer & Jensen 1989 [23], while vitamin C content was determined according to the method described in Romeu-Nadal et al., 2006 [24], and vitamin B complex content was determined according to the method described in Batifoulier, et al., 2005 [25].

### Animals

Forty-eight male albino rats were used, weighting 130 ± 10 g at the start of the experiments. Prior to the studies, the animals were randomly and assigned to treatment groups. Four rats were housed per cage (size 26 × 41 cm) and placed in the experimental room for acclimatization 24 h before the test. The animals were fed with standard laboratory diet and with tap water ad libitum, and kept in an air-conditioned animal room at 23 ± 1 °C with a 12 h light/dark cycle. Animal care and handling was performed in conformance with approved protocols of Cairo University and Egyptian Community guidelines for animal care.

### Experimental induction of obesity by HFD

Control rats received a normal pellet diet (NPD) for three months the remaining rats received HFD to establish diet-induced obesity. It provides 58 % fat, 25 % protein and 17 % carbohydrate, as a percentage of total kcal. The composition and preparation of HFD was described by Srinivasan et al., 2004 [26] as the following components (g/kg): powdered NPD, 365 (Egyptian market); lard, 310 (Egyptian market); casein, 250 (Difco, Becton Dickinson, France); cholesterol, 10 (Oxford Lab, Mumbai, India); vitamin and mineral mix, 60 (Sigma–Aldrich, MO, USA; 8ADWIC Co., Cairo, Egypt); DL-methionine, 3.0 (Sigma–Aldrich, MO, USA; 8 ADWIC Co., Cairo, Egypt); Yeast powder, 1.0 (Egyptian market), sodium chloride, 1.0 (Egyptian market).

### Experimental design

Rats were randomly allocated into eight groups of six animals each:

- Group 1: normal control group fed NPD.
- Group 2 (HFD): rats received HFD for 3 month.
- Group 3 (SD): rats received HFD for 2 month and returned to NPD for 1 month.
- Group 4 (T 200): rats received HFD for 2 month, then returned to NPD and treated with tomato extract 200 mg/kg/day, p.o. for 1 month.
- Group 5 (T 400): rats received HFD for 2 month, then returned to NPD and treated with tomato extract 400 mg/kg/day, p.o. for 1 month.
- Group 6 (B 200): rats received HFD for 2 month, then returned to NPD and treated with broccoli extract 200 mg/kg/day, p.o. for 1 month.
- Group 7 (B 400): rats received HFD for 2 month, then returned to NPD and treated with broccoli extract 400 mg/kg/day, p.o. for 1 month.
- Group 8 (CX): rats received HFD for 2 month, then returned to NPD and treated with Chromax 400 μg/kg/day, p.o. for 1 month.

### Drugs

Chromax (Eva Pharma, Egypt) was dissolved in distilled water and administered orally.

### Toxicity study

The toxicity of aqueous extracts of tomato and broccoli were tested using four doses (200, 400, 1000 and 2000 mg/kg) (six rats for each dose). Six control rats were kept under the same conditions without any treatments. The animals were observed continuously during the first hour, and then every hour for 6 h, then after 12 and 24 h, and finally after every 24 h, up to 3 weeks, for mortality or any physical signs of toxicity such as writhing, gasping, salivation, diarrhea, cyanosis, pupil size, any nervous manifestations.

### Blood samples and biochemical analysis

At the end of the study, rats were fasted overnight, anesthetized with thiopental sodium (50 mg/kg) [27], and blood samples were collected from retinal vein (5 ml per rat). Blood samples were centrifuged at 3000 rpm for 15–30 min after collection and stored at −80 °C until analyzed. Serum glucose level was estimated enzymatically using TECHNICAL BULLETIN Sigma Aldrich (USA), and triglyceride levels (TGs) were estimated using enzymatic assay kit from Xpress Bio Life Science Products. Total cholesterol (TC), low-density lipoprotein cholesterol (LDL-C) and high-density lipoprotein cholesterol (HDL-C) were measured colorimetrically using assay kits from Biochain (Hayward, USA) according to the manufactured instructions.

### ELISA

Serum insulin, resistin, leptin, and adiponectin were determined using the corresponding rat enzyme immunoassay kits (insulin: SPI bio, Downers Grove, France, resistin: Biovendor research and diagnostic products, leptin: RayBiotech, Inc., Norcross GA, USA, adiponectin: Chemicon international, USA, and AST: Shanghai Sunred Biological Technology Co., China) using an ELISA microplate reader Sunrise, TECAN, Austria.

### Calculation of insulin sensitivity, resistance, adipose tissue index

Insulin resistance was determined using the homeostasis model assessment index for insulin resistance (HOMA-IR) using the following formula: HOMA-IR index = [fasting glucose (mg/dl) * fasting insulin (μU/ml)]/405). To assess insulin sensitivity, the revised quantitative insulin sensitivity check index (R-QUICKI) = 1/[log fasting insulin (μU/ml) + log fasting glucose (mg/dl)] was used [28, 29]. Additionally, the adipose tissue index (API = retroperitoneal adipose tissue X100) was calculated.

### Statistical analyses

All data were expressed as mean ± standard error of mean (SEM) and analyzed using prism program version 6. For all parameters, comparisons among groups were carried out using one-way analysis of variance (ANOVA) followed by Bonferroni’s multiple comparisons test. All *P* values reported are two-tailed and *P* ‹ 0.05 was considered significant.

## Results

### Toxicity study

The toxicity study revealed the non-toxic nature of aqueous extracts of tomato and broccoli at doses up to 2 gm/kg. No deaths were reported, and the rats did not show any drug-induced physical signs of toxicity during the whole experiment period.

### Effect of tomato and broccoli extract on blood glucose, insulin and insulin resistance markers

The results of the experiments are summarized in Table 1. Mean serum levels of insulin and glucose were significantly increased in the HFD and SD groups compared to the control group (*P* value < 0.0001). On the other hand, the mean serum levels of insulin and glucose were significantly reduced in the T 200, T 400, B 200, B 400 and CX groups when compared to the HFD and SD groups *(P* value < 0.0001). The mean serum level of insulin was significantly reduced in the T 400 group compared to the B 400 and CX groups (*P* value < 0.001) while glucose serum level was significantly reduced in the T 400 group compared to the B 400 group (*P* value < 0.01). No significant difference of glucose serum level was found between the T 400 and CX groups.

**Table 1:** Effect of tomato and broccoli extract on serum insulin, glucose, on calculated HOMA- IR and R-QUICKI in HFD induced obesity in rats

Groups	Serum insulin (uIU/ml)	Serum glucose (mg/dl)	HOMA-IR	R-QUICKI
Control	2.52 ± 0.28	67.8 ± 3.23	0.43 ± 0.06	0.455 ± 0.01
HFD	44.1 ± 2.14*	169 ± 1.38*	18.5 ± 0.96*	0.25 ± 0.004*
SD	30.9 ± 1.28*^,^**	156 ± 1.86*^,^**	11.9 ± 0.41*^,^**	0.27 ± 0.004*^,^**
T 200	12.1 ± 0.75*^,^**^,^***	138 ± 3.82*^,^**^,^***	4.15 ± 0.34*^,^**^,^***	0.31 ± 0.008*^,^**^,^***
T 400	6.72 ± 0.36*^,^**^,^***	99.3 ± 4.0*^,^**^,^***	1.66 ± 0.15*^,^**^,^***	0.35 ± 0.01*^,^**^,^***
B 200	19 ± 0.71*^,^**^,^***	136 ± 3.0*^,^**^,^***	6.39 ± 0.22*^,^**^,^***	0.29 ± 0.004*^,^**^,^***
B 400	9.38 ± 0.47*^,^**^,^***^,^****	115 ± 2.96*^,^**^,^***^,^****	2.67 ± 0.18*^,^**^,^***^,^****	0.33 ± 0.008*^,^**^,^***^,^****
CX	9.48 ± 0.53*^,^**^,^***^,^****	108 ± 2.79*^,^**^,^***	2.54 ± 0.2*^,^**^,^***^,^****	0.33 ± 0.01*^,^**^,^***^,^****

Tomato and broccoli extracts treatment at different doses significantly reduced serum levels of insulin and glucose in HFD rats. These extracts also significantly reduced HOMA-IR increased R- QUICKI. C = control; HFD = high fat diet; SD = stop diet; T 200 = tomato extract 200 mg/kg; T 400 = tomato 400 mg/kg; B 200 = broccoli 200 mg/kg; B 400 = broccoli 400 mg/kg; CX = Chromax. HOMA-IR = Homeostasis model assessment index for insulin resistance; R-QUICKI = revised quantitative insulin sensitivity check index. Results were expressed as mean ± SEM and analyzed using one-way ANOVA followed by Bonferroni’s post hoc test. *Significant from control at *P* < 0.0001, **Significant from HFD at *P* < 0.0001, ***Significant from SD at *P* < 0.0001, ****Significant from T 400 at *P* < 0.005. Results were expressed as mean ± SEM and analyzed using one-way ANOVA followed by Bonferroni’s post hoc test

The calculated HOMA-IR was significantly increased while R-QUICKI was significantly reduced in the HFD and SD groups compared to the control group (*P* value < 0.0001). HOMA-IR values were significantly reduced while R-QUICKI values were significantly increased in the T 200, T 400, B 200, B 400 and CX groups when compared to the HFD and SD groups (*P* value < 0.0001). HOMA-IR values were also significantly reduced while R-QUICKI was also significantly increased in the T 400 group compared to the B 400 and CX groups (*P* value was < 0.05), (Table 1).

### Effect of tomato and broccoli extract on body weight and adipose tissue index

The % weight increase was significantly larger in the HFD and SD groups compared to the control group (*P* value < 0.0001). However, the % weight increase was significantly reduced in the SD, T 200, T 400, B200, B 400 and CX groups compared to the HFD group (*P* value < 0.001). The API was significantly increased in the HFD and SD groups compared to the control group (*P* value was < 0.0001). In contrast, the API was significantly reduced in the T 200, T 400, B200, B 400 and CX groups compared to the HFD group (*P* value < 0.001). The difference in the API between the T 400 and B 400 groups compared to the control group was not significant (Table 2).

**Table 2:** Effect of tomato and broccoli extract on body weight and adipose tissue index in HFD induced obesity in rats

Groups	Initial weight (gm)	Final weight (gm)	% Δ weight (gm)	Adipose tissue index
Control	127 ± 1.59	166 ± 5.4	30.3 ± 2.89	0.82 ± 0.07
HFD	126 ± 1.49	259 ± 6.73	106 ± 5.13*	2.6 ± 0.21*
SD	126 ± 1.31	218 ± 5.36	72.8 ± 4.79*^,^**	2.21 ± 0.13*^,^**
T 200	128 ± 1.03	199 ± 7.39	56.1 ± 6.11*^,^**^,^***	1.1 ± 0.04*^,^**^,^***
T 400	126 ± 1.59	190 ± 4.54	51.6 ± 4.11*^,^**^,^***	0.81 ± 0.08**^,^***
B 200	133 ± 1.06	214 ± 5.68	60.4 ± 4.81*^,^**	1.47 ± 0.18*^,^**^,^***
B 400	132 ± 1.53	211 ± 9.12	59.1 ± 6.45*^,^**	1.03 ± 0.1**^,^***
CX	136 ± 1.28	221 ± 4.49	60.2 ± 3.19*^,^**^,^***	1.58 ± 0.27*^,^**

Tomato and broccoli extracts treatment at different doses significantly reduced body weight and adipose tissue index in HFD rats. C = control; HFD = high fat diet; SD = stop diet; T 200 = tomato extract 200 mg/kg; T 400 = tomato 400 mg/kg; B 200 = broccoli 200 mg/kg; B 400 = broccoli 400 mg/kg; CX = Chromax. Results were expressed as mean ± SEM and analyzed using one-way ANOVA followed by Bonferroni’s post hoc test. * = Significant from control at *P* < 0.0001, **= Significant from HFD at *P* < 0.0001, *** = Significant from SD at *P* < 0.05. Results were expressed as mean ± SEM and analyzed using one-way ANOVA followed by Bonferroni’s post hoc test

### Effect of tomato and broccoli extract on serum lipid profile

Mean serum level of total cholesterol, triglycerides and LDL-cholesterol were significantly increased in the HFD group compared to the control group (*P* value < 0.0001). The serum level of total cholesterol, triglycerides and LDL-cholesterol were significantly reduced in T 200, T 400, B 200, B 400 and CX groups compared to HFD group (*P* value < 0.0001). Although total cholesterol, LDL, and triglycerides were significantly reduced in T 400 group compared to B 400 with no significant difference between T 400 group and CX group, LDL-cholesterol was significantly reduced in T 400 group compared to B 400 and CX groups (*P* value < 0.001).

HDL-cholesterol levels were significantly reduced in the HFD group compared to the control group (*P* value was < 0.0001). The mean serum levels of HDL-cholesterol were significantly increased in the T 200, T 400, B 200, B 400 and CX groups compared to the HFD group (*P* value < 0.0001). No significant difference was found between the T 400, B 400 and CX groups. There was also no significant difference between the control and SD groups in lipid profile (Table 3).

**Table 3:** Effect of tomato and broccoli extract on lipid profile in HFD induced obesity in rats

Groups	TC (mg/dl)	TAG (mg/dl)	LDL-C (mg/dl)	HDL-C (mg/dl)
Control	53.7 ± 1.76	40 ± 1.06	19.3 ± 0.55	33.5 ± 0.76
HFD	147 ± 3.66*	145 ± 3.68*	102 ± 2.25*	14.5 ± 0.22*
SD	55.5 ± 1.95**	40.7 ± 1.02**	19.7 ± 0.71**	34.5 ± 0.76**
T 200	85.5 ± 1.57*^,^**^,^***	79.5 ± 4.19*^,^**^,^***	48.3 ± 1.15*^,^**^,^***	23.7 ± 0.21*^,^**^,^***
T 400	62.3 ± 1.15*^,^**^,^***	51 ± 0.85*^,^**^,^***	27 ± 0.57*^,^**^,^***	24.5 ± 0.22*^,^**^,^***
B 200	121 ± 2.62*^,^**^,^***	127 ± 1.82*^,^**^,^***	73.5 ± 3.14*^,^**^,^***	18 ± 0.36*^,^**^,^***
B 400	84.2 ± 2.5*^,^**^,^***^,^****	73.7 ± 1.8*^,^**^,^***^,^****	48 ± 1.06*^,^**^,^***^,^****	23.3 ± 0.61*^,^**^,^***
CX	63 ± 1.97*^,^**^,^***	50 ± 1.88*^,^**^,^***	31.2 ± 0.6*^,^**^,^***^,^****	25.5 ± 0.22*^,^**^,^***

Tomato and broccoli extracts treatment at different doses significantly reduced lipid profile parameters in HFD rats. C = control; HFD = high fat diet; SD = stop diet; T 200 = tomato extract 200 mg/kg; T 400 = tomato 400 mg/kg; B 200 = broccoli 200 mg/kg; B 400 = broccoli 400 mg/kg; CX = Chromax. TC = total cholesterol; TAG = triglycerides; LDL-C = low-density lipoprotein cholesterol; HDL-C = high-density lipoprotein cholesterol. Results were expressed as mean ± SEM and analyzed using one-way ANOVA followed by Bonferroni’s post hoc test. * =Significant from control at *P* < 0.0001, **Significant from HFD at *P* < 0.0001, *** =Significant from SD at *P* < 0.05, **** = Significant from T 400 at *P* < 0.005. Results were expressed as mean ± SEM and analyzed using one-way ANOVA followed by Bonferroni’s post hoc test

### Effect of tomato and broccoli extract on serum resistin, leptin and adiponectin

**Fig. 1:**
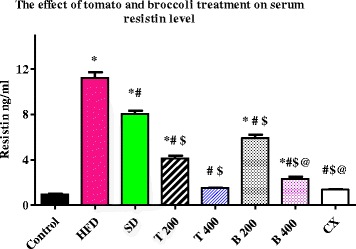
Serum level of resistin (ng/ml) in the experimental groups. Tomato and broccoli extracts reduced serum level of resistin in the HFD rats at the end of 1 month treatment; C = control; HFD = high fat diet; SD = stop diet; T 200 = tomato extract 200 mg/kg; T 400 = tomato 400 mg/kg; B 200 = broccoli 200 mg/kg; B 400 = broccoli 400 mg/kg; CX = Chromax. Results were expressed as mean ± SEM. and analyzed using one-way ANOVA followed by Bonferroni’s post hoc test.* = Significant from control at *P* < 0.0001, # = Significant from HFD at *P* < 0.0001, $ = Significant from SD at *P* < 0.000, @ = Significant from T 400 at *P* < 0.001

**Fig. 2:**
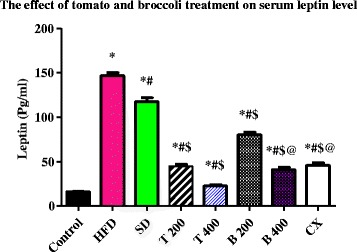
Serum level of leptin (pg/ml) in the experimental groups. Tomato and broccoli extracts reduced serum level of leptin in the HFD rats at the end of 1 month treatment; C = control; HFD = high fat diet; SD = stop diet; T 200 = tomato extract 200 mg/kg; T 400 = tomato 400 mg/kg; B 200 = broccoli 200 mg/kg; B 400 = broccoli 400 mg/kg; CX = Chromax. Results were expressed as mean ± SEM and analyzed using one-way ANOVA followed by Bonferroni’s post hoc test. * = Significant from control at *P* < 0.0001, # = Significant from HFD at *P* < 0.0001, $ = Significant from SD at *P* < 0.0001, @ == Significant from T 400 at *P* < 0.001

**Fig. 3:**
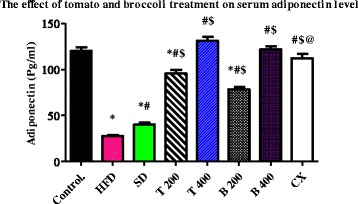
Serum level of adiponectin (pg/ml) in the experimental groups. Tomato and broccoli extracts increased serum levels of adiponectin in the HFD rats at the end of 1 month treatment; C = control; HFD = high fat diet; SD = stop diet; T 200 = tomato extract 200 mg/kg; T 400 = tomato 400 mg/kg; B 200 = broccoli 200 mg/kg; B 400 = broccoli 400 mg/kg; CX = Chromax. Results were expressed as mean ± SEM and analyzed using one-way ANOVA followed by Bonferroni’s post hoc test. * = Significant from control at *P* < 0.0001, # = Significant from HFD at *P* < 0.0001, $ = Significant from SD at *P* < 0.0001, @ = Significant from T 400 at *P* < 0.001

The mean serum level of resistin and leptin were significantly increased in the HFD and SD groups compared to the control group (*P* < 0.0001). Serum levels of resistin and leptin were significantly reduced in the T 200, T 400, B 200, B 400 and CX groups compared to the HFD and SD groups (*P* < 0.0001). Also, serum levels of resistin and leptin were significantly reduced in the T 400 group compared to the B 400 and CX groups (*P* < 0.001). Adiponectin levels were significantly decreased in the HFD and SD groups compared to the control group (*P* < 0.0001). The mean serum level of adiponectin was significantly increased in the T 200, T 400, B 200, B 400 and CX groups compared to the HFD and SD groups (*P* < 0.0001). Serum adiponectin levels were significantly increased in the T 400 group compared to the CX group (*P* value < 0.001). No significant difference in serum adiponectin levels was found between the control and the T 400, B 400, and CX groups (Table 4 and Figs. 1, 2, and 3) Additional file 1.

**Table 4:** Effect of tomato and broccoli extract on serum resistin, leptin and adiponectin in HFD induced obesity in rats

Groups	Resistin (ng/ml)	Leptin (Pg/ml)	Adiponectin (Pg/ml)
Control	0.93 ± 0.05	16.3 ± 0.57	120 ± 3.81
HFD	11.2 ± 0.48*	147 ± 3.70*	27.8 ± 0.94*
SD	8.05 ± 0.29*^,^**	118 ± 4.20*^,^**	40.2 ± 2.36*^,^**
T 200	4.13 ± 0.22*^,^**^,^***	44.8 ± 2.13*^,^**^,^***	95.5 ± 3.84*^,^**^,^***
T 400	1.51 ± 0.04*^,^**^,^***	22.7 ± 0.84*^,^**^,^***	131 ± 3.84*^,^**
B 200	5.91 ± 0.29*^,^**^,^***	80.3 ± 3.14*^,^**^,^***	78.5 ± 2.42*^,^**^,^***
B 400	2.32 ± 0.15*^,^**^,^***^,^****	41 ± 2.45*^,^**^,^***^,^****	122 ± 3.61*^,^**
CX	1.37 ± 0.03*^,^**^,^***^,^****	45.7 ± 2.91*^,^**^,^***^,^****	112 ± 4.77**^,^***^,^****

Tomato and broccoli extracts treatment at different doses significantly reduced serum resistin and leptin while increased adiponectin levels in HFD rats. C = control; HFD = high fat diet; SD = stop diet; T 200 = tomato extract 200 mg/kg; T 400 = tomato 400 mg/kg; B 200 = broccoli 200 mg/kg; B 400 = broccoli 400 mg/kg; CX = Chromax

*= Significant from control at *P* < 0.0001, **Significant from HFD at *P* < 0.0001, ***Significant from SD at *P* < 0.05, ****Significant from T 400 at *P* < 0.001. Results were expressed as mean ± SEM and analyzed using one-way ANOVA followed by Bonferroni’s post hoc test

### Nutritional value of tomato and broccoli

Table 5 shows the proximate analysis, vitamin and mineral contents of the fresh samples of tomato (*S. lycopersicum L*.) and broccoli (*B. oleracea L. Italica*) grown in Egypt that were used to make the extracts used in this study.

**Table 5:** Proximate analysis, vitamins contents and minerals contents of the fresh samples of tomato (*S. lycopersicum* L.) and broccoli (*B. oleracea L. Italica)*

Item		Tomato	Broccoli
Proximate analysis	Nutritive Value	24.32 Kcal	41.76 Kcal
		Percentage/Fresh weight	
	Moisture content	93.7 %	88.6 %
	Total ash	0.46 %	0.87 %
	Total carbohydrates	4.2 %	6.9 %
	Total lipids	0.44 %	0.32 %
	Total proteins	0.89 %	2.82 %
Vitamins		Amount in 100 gm fresh weight	
	Vitamin A (IU)	837	624
	Vitamin E (mg)	0.539	0.36
	Vitamin C (mg)	24.68	90.02
	Vitamin B1 (mg)	0.057	0.121
	Vitamin B2 (mg)	0.036	0.175
	Vitamin B3 (mg)	0.54	0.69
	Vitamin B6 (mg)	0.007	0.28
	Vitamin B12 (mg)	ND^a^	ND^a^
Minerals	Potassium (mg)	247.84	317.48
	Calcium (mg)	15.98	48.07
	Sodium (mg)	73.6	33.96
	Chromium (mg)	0.16	0.02
	Copper (mg)	0.077	0.029
	Manganese (mg)	0.19	0.24
	Zinc (mg)	0.21	0.44

^a^ND: Not detected

## Discussion

Obesity is becoming a global threat to human health. A sedentary life style and change of diet are implicated in the development of obesity [30]. Diabesity is a term that expresses the association between obesity and type II diabetes [31]. A high fat diet in rats has been shown to induce metabolic syndrome similar to type II diabetes and insulin resistance [3]. Since there is a great effort among researchers to identify dietary components that prevent or treat obesity complications, we investigated the possible role of tomato and broccoli extract on glucose homeostasis in HFD-induced obesity in rats. This study reports for the first time that both broccoli and tomato extracts improved glucose homeostasis in HFD-induced obesity rats, possibly through the reduction of resistin in addition to their high nutritional value.

Tomato and broccoli extracts significantly reduced serum level of glucose after one month of treatment in HFD-induced obesity rats. Tomato and broccoli treatment also improved the lipid profile, the API, as well as adiponectin and leptin levels in HFD-induced obesity rats of this study. Tomato extract at a concentration of 400 mg/kg was more effective in reducing glucose and leptin and increasing adiponectin compared with broccoli and Chromax®, the commercially used chromium supplement. This hypoglycemic hypolipidemic effect can be attributed to improvement of whole body insulin resistance [32].

To explain the mechanism by which these extracts may work, we measured serum resistin in our rat model of diabesity. Our results shows that treatment with tomato and broccoli extracts significantly reduced serum level of resistin compared to HFD group and SD group (*P* value < 0.0001). Both tomato and broccoli extracts had similar effects on serum resistin levels as Chromax®, as there was a non-significant difference was found between the T 400, B 400 and CX groups (Table and Fig. 1). These results could in part explain the hypoglycemic effect of tomato and broccoli extracts observed in our study. Resistin is an adipocyte hormone that participates in the regulation of glucose metabolism, increasing the blood glucose levels and hepatic glucose production. Elevated levels of resistin are postulated to cause insulin resistance [4]. Our results indeed support the role of resistin in the maintenance of glucose homeostasis via a resistin-mediated mechanism.

Our results are in agreement with many researchers who have found beneficial effects of tomato in obesity and the related metabolic syndrome. Ghavipour et al.*,* [33] found that tomato juice administered to overweight and obese women reduced the production of inflammatory cytokines. Also Ghorbani et al. [34], observed antioxidant effect for tomato juice in overweight and obese women. To our knowledge this is the first report of the effect of tomato extract on serum resistin level [4].

The observed effect of tomato extract in our study could be attributed to the antioxidant effect of lycopene (the most effective anti-oxidant of the carotenoids in tomatoes). Kim et al., 2015 [35] found that tomato extract potentially suppresses inflammation by inhibiting the production of NO or pro-inflammatory cytokines during the interaction between adipocytes and macrophages in cell culture. Since obesity is a chronic inflammatory condition and the inflammation is considered a source of oxidative stress, so lycopene can be useful in this condition [34].

Broccoli extract appears to have similar beneficial effects on glucose homeostasis as tomato extract. Although tomato extract had more significant effect in correcting glucose and insulin level (Table 1) at the dose of 400 mg/kg, both had almost the same effect on serum resistin level (Table 4 and Fig. 3). Broccoli may exert its beneficial effect on glucose homeostasis via its well-documented anti-oxidant effect [11, 36–39]. Also, Christensen et al., 2014 [14], found that broccoli extract enhances glucose uptake in porcine cell culture and stimulated the differentiation of adipocytes in C. *Elegans*. These findings may explain in part the beneficial effect of broccoli extracts observed in our study.

The beneficial effects of both tomato and broccoli extract on glucose and lipid homeostasis found here could be also explained by the fact that both extract are rich in chromium ([40] http://www.eufic.org/). Chromium is a trace element that was found to have beneficial effects on human health. Several studies have found that chromium supplementation has decreased blood glucose, decreased blood cholesterol and triglyceride, or decreased insulin requirements in both diabetic patients and diabetic animals [41, 42]. In fact, the similarity of the effects of both tomato and broccoli extracts to the effect of Chromax® on insulin and glucose levels in HFD rats suggest that their effects may be acting through the same mechanism. Although the exact mechanism through which chromium supplements act to increase insulin sensitivity is still unclear, it has been suggested that it may act through the stimulation of tyrosine kinase activity in insulin sensitive cells as well as anti-inflammatory effects [41, 43–46].

**Fig. 4:**
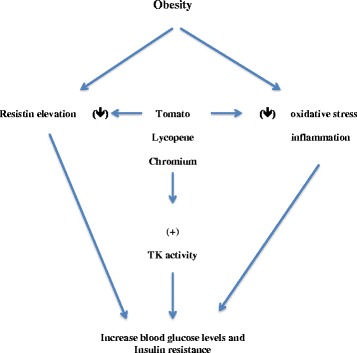
A schematic representation of possible mechanisms by which tomato extract can regulate blood glucose level in obesity. TK = tyrosine kinase

We propose that lycopene of tomato extract reduces oxidative stress due to inflammation associated with obesity and consequently reduces resistin and other associated adipocyte production. This in turn corrects glucose and insulin levels in obesity and restores glucose homeostasis. Also chromium in both tomato and broccoli extracts may stimulate tyrosine kinase activity in glucose sensitive cells in addition to its potential anti-inflammatory effects that may enhance glucose uptake and maintain glucose homeostasis. This theory may explain why tomato extract was more effective in reducing blood glucose and insulin levels due to the synergistic effects of lycopene and chromium in tomato extract (Fig. 4).

In addition to the above-mentioned positive effects of tomato and broccoli extracts, these vegetables grown in Egypt also have high nutritional values (please review Table 5). This strongly supports their use as functional foods.

## Conclusions

In conclusion, our results shows that tomato and broccoli extract one month treatment reduces serum resistin and restores insulin sensitivity in HFD rats. Tomato extract was more effective than broccoli in reducing blood glucose levels, however, further studies are required to assess the potential use of these extract as a treatment for type II diabetes in human and to determine its effective doses, as well as its active ingredients. Our study also recommends the use of both tomato and broccoli as a functional food with both nutritional and therapeutic values.

## Additional file

Additional file 1: Individual data for serum resistin, leptin, and adiponectin. (DOCX 18 kb)
