# Supplementary material for: Resistin mediates tomato and broccoli extract effects on glucose homeostasis in high fat diet-induced obesity in rats
Source: BMC Complement Altern Med. 2016 Jul 18;16:225. doi: 10.1186/s12906-016-1203-0 (PMC4949892; doi:10.1186/s12906-016-1203-0)
Supplement: Additional file 1: — Individual data for serum resistin, leptin, and adiponectin. (DOCX 18 kb) [file 12906_2016_1203_MOESM1_ESM.docx]

**Individual data of serum Resistin**

| **Control** | **HFD** | **SD** | **T200** | **T400** | **B200** | **B400** | **CX** |
| --- | --- | --- | --- | --- | --- | --- | --- |
| 1.11 | 10.6 | 7.98 | 4.11 | 1.64 | 6.15 | 2.87 | 1.29 |
| 0.98 | 13.1 | 8.42 | 3.98 | 1.59 | 5.74 | 2.61 | 1.34 |
| 0.87 | 12.0 | 6.98 | 5.11 | 1.34 | 4.97 | 1.98 | 1.51 |
| 1.04 | 11.3 | 9.11 | 3.51 | 1.52 | 5.27 | 2.45 | 1.37 |
| 0.79 | 9.8 | 7.66 | 4.29 | 1.47 | 6.74 | 1.87 | 1.32 |
| 0.83 | 10.5 | 8.12 | 3.77 | 1.51 | 6.59 | 2.16 | 1.36 |

**Individual data of serum Leptin**

| **Control** | **HFD** | **SD** | **T200** | **T400** | **B200** | **B400** | **CX** |
| --- | --- | --- | --- | --- | --- | --- | --- |
| 16.2 | 142 | 100.7 | 44.1 | 22.4 | 86.2 | 43.6 | 40.3 |
| 17.6 | 138 | 121.1 | 39.6 | 19.8 | 90.2 | 36.2 | 36.8 |
| 18.4 | 152 | 118.9 | 52.1 | 20.9 | 69.7 | 43.2 | 41.3 |
| 14.9 | 149 | 129.4 | 38.7 | 23.6 | 74.1 | 33.2 | 49.1 |
| 15.2 | 161 | 111.2 | 45.6 | 25.1 | 83.2 | 50.1 | 52.4 |
| 15.6 | 138 | 124.6 | 48.9 | 24.5 | 78.6 | 39.8 | 54.1 |

**Individual data of serum Adiponectin**

| **Control** | **HFD** | **SD** | **T200** | **T400** | **B200** | **B400** | **CX** |
| --- | --- | --- | --- | --- | --- | --- | --- |
| 111 | 31 | 36 | 95 | 137 | 79 | 132 | 118 |
| 124 | 25 | 44 | 104 | 118 | 81 | 128 | 131 |
| 131 | 26 | 39 | 78 | 139 | 76 | 116 | 102 |
| 109 | 27 | 50 | 98 | 121 | 87 | 126 | 116 |
| 116 | 28 | 37 | 103 | 134 | 69 | 108 | 101 |
| 129 | 30 | 35 | 95 | 139 | 79 | 119 | 105 |
